# Advancing Key Gaps in the Knowledge of *Plasmodium vivax* Cryptic Infections Using Humanized Mouse Models and Organs-on-Chips

**DOI:** 10.3389/fcimb.2022.920204

**Published:** 2022-07-04

**Authors:** Iris Aparici Herraiz, Hugo R. Caires, Óscar Castillo-Fernández, Núria Sima, Lourdes Méndez-Mora, Ruth M. Risueño, Jetsumon Sattabongkot, Wanlapa Roobsoong, Aurora Hernández-Machado, Carmen Fernandez-Becerra, Cristina C. Barrias, Hernando A. del Portillo

**Affiliations:** ^1^ Barcelona Institute for Global Health (ISGlobal), Hospital Clínic - Universitat de Barcelona, Barcelona, Spain; ^2^ Institut d’Investigació en Ciències de la Salut Germans Trias i Pujol, Badalona, Spain; ^3^ i3S - Instituto de Investigação e Inovação em Saúde, Universidade do Porto, Porto, Portugal; ^4^ Institute of Nanoscience and Nanotechnology (IN2UB), University of Barcelona, Barcelona, Spain; ^5^ Department of Condensed Matter Physics, University of Barcelona (UB), Barcelona, Spain; ^6^ Josep Carreras Leukaemia Research Institute (IJC), Barcelona, Spain; ^7^ Mahidol Vivax Research Unit, Faculty of Tropical Medicine, Mahidol University, Bangkok, Thailand; ^8^ Centre de Recerca Matemàtica (CRM), Barcelona, Spain; ^9^ INEB – Instituto de Engenharia Biomédica, Universidade do Porto, Porto, Portugal; ^10^ ICBAS – Instituto de Ciências Biomédicas de Abel Salazar, Universidade do Porto, Porto, Portugal; ^11^ Institució Catalana de Recerca i Estudis Avançats (ICREA), Barcelona, Spain

**Keywords:** humanized mouse, organs-on-a-chip, *Plasmodium vivax*, models, Key gaps in the knowledge

## Abstract

*Plasmodium vivax* is the most widely distributed human malaria parasite representing 36.3% of disease burden in the South-East Asia region and the most predominant species in the region of the Americas. Recent estimates indicate that 3.3 billion of people are under risk of infection with circa 7 million clinical cases reported each year. This burden is certainly underestimated as the vast majority of chronic infections are asymptomatic. For centuries, it has been widely accepted that the only source of cryptic parasites is the liver dormant stages known as hypnozoites. However, recent evidence indicates that niches outside the liver, in particular in the spleen and the bone marrow, can represent a major source of cryptic chronic erythrocytic infections. The origin of such chronic infections is highly controversial as many key knowledge gaps remain unanswered. Yet, as parasites in these niches seem to be sheltered from immune response and antimalarial drugs, research on this area should be reinforced if elimination of malaria is to be achieved. Due to ethical and technical considerations, working with the liver, bone marrow and spleen from natural infections is very difficult. Recent advances in the development of humanized mouse models and organs-on-a-chip models, offer novel technological frontiers to study human diseases, vaccine validation and drug discovery. Here, we review current data of these frontier technologies in malaria, highlighting major challenges ahead to study *P. vivax* cryptic niches, which perpetuate transmission and burden.

## Background

Human malaria is a disease caused by five species of plasmodia of which *Plasmodium falciparum* and *Plasmodium vivax* represent the vast majority of burden whereas *Plasmodium ovale*, *Plasmodium malaria* and the recent zoonosis caused by *Plasmodium knowlesi* in South East Asia, are likely to contribute to less than 5% of such burden (Battle et al., 2019). In the particular case of *P. vivax*, its burden has dramatically decreased from 11.9-22 million cases in 2013 to 7 million clinical cases in 2020 (WHO, 2020). This panorama would indicate that elimination of *P. vivax* can be achieved with the present control tools available. However, in low-endemic countries that have been targeting vivax malaria for elimination, an increasing number of asymptomatic infections capable of transmitting to mosquitoes are being reported (Cheng et al., 2015). In fact, during chronic infections, numerous epidemiological field studies support that >90% of infections are sub-microscopic and asymptomatic (Angrisano and Robinson, 2022). Thus, the global burden of this species is certainly underestimated. Moreover, experts agree that *P. vivax* will be the last human malaria parasite species to be eliminated due to its unique biology, including (i) the presence of hypnozoites, a latent (dormant) form of the parasite that develops in the liver (Krotoski, 1985) which can reactivate weeks or months after the primary infection causing clinical relapses (White, 2011); (ii) the limitation of using primaquine and tafenoquine to kill hypnozoites in pregnant women and G6PD-defficient individuals due to the risk of acute hemolytic anemia (Baird, 2019); (iii) the risk of primaquine treatment failure in patients with particular cytochrome P450 isozyme 2D6 (CYP2D6) polymorphisms (Suarez-Kurtz, 2021); (iv) the recent findings that invasion of merozoites into reticulocytes is not limited to the Duffy binding protein (Ménard et al., 2010) and the detection of *P. vivax* in sub-Saharan Africa, where this blood group is nearly absent (Zimmerman, 2017); (v) the outdoor biting behaviour of vectors transmitting *P. vivax*, that results on low efficacy control measures when impregnated bed nets are used. All together, these unique aspects of the biology of *P. vivax* strongly support the generalized view that this species will be the last human malaria parasite to be eliminated (Mueller et al., 2009).

**Figure 1:**
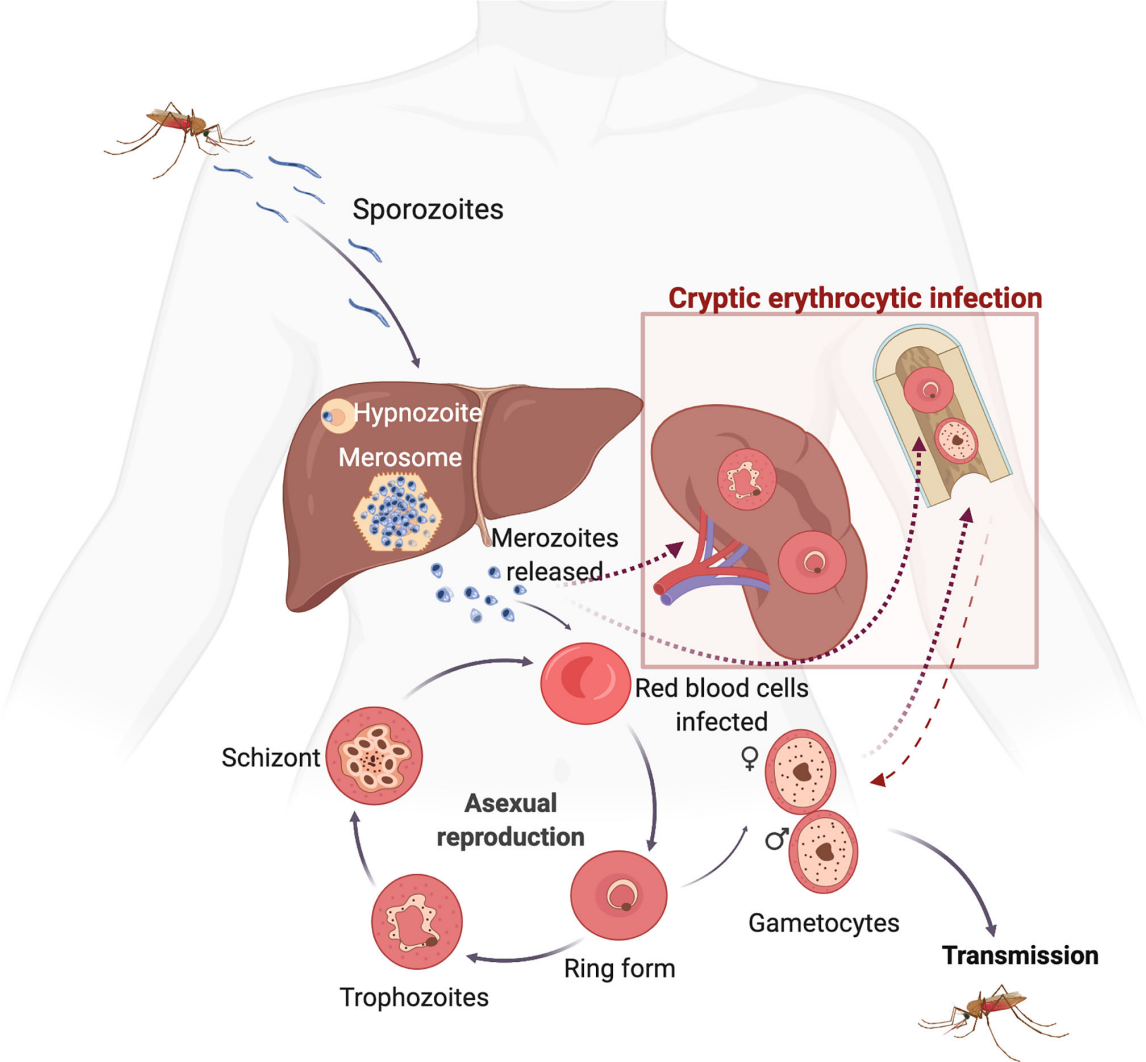
Life cycle of *Plasmodium vivax* highlighting new cryptic erythrocytic stages. During a blood meal, malaria-infected mosquitos inject sporozoites which after reaching the bloodstream enter hepatocytes initiating the pre-erythrocytic cycle. Within the liver, *P. vivax* either differentiates (i) into a dormant stage called a hypnozoite which, upon reactivation, causes clinical relapses, or (ii) into tissue schizonts, which after thousands of mitotic replications in membranous sacks known as merosomes, release merozoites into the bloodstream initiating the erythrocytic cycle. In this cycle, *P. vivax* merozoites predominantly, if not exclusively, invade reticulocytes starting asexual blood stage differentiation of rings, trophozoites, schizonts and egress to invade new red blood cells. This cyclical developmental process takes about 48 h. In addition, *P. vivax* produces specific proteins to create caveola–vesicle complexes that appear as profuse speckling in Giemsa-stained blood smears, known as Schüfnner’s dots. Moreover, some *P. vivax* parasites can differentiate into mature gametocytes before a clinical infection and illness develops, thus having the advantage of continued transmission to the insect vector before the appearance of clinical symptoms and subsequent treatment. Remarkably, presence of parasites in the spleen and the bone marrow represents novel cryptic erythrocytic infections that need to be incorporated in the life cycle of this species (boxed). Infections of these organs can be either directly from invasion of merozoites into the reticulocyte-rich bone marrow and spleen, or by infected-reticulocytes in peripheral blood. Circulating gametocytes are rounded shape and on uptake in the blood meal of anopheles mosquitoes begin the sexual cycle. This includes release of the male and female gametes, fertilisation, and formation of a motile ookinete that crosses the midgut epithelium. Differentiation into a new replicative form known as the oocyst, release of sporozoites, migration, and invasion of the salivary glands ends this complex life cycle in which the parasite undergoes more than ten stages of cellular differentiation and invades at least four types of cells within two different hosts. (Created with BioRender.com by Carmen Fernandez-Becerra).

Cryptic stages can be defined as parasites difficult to detect with currently available tools which persist in different host tissues during chronic asymptomatic infections. For centuries, it was amply accepted that the only source of cryptic stages in *P. vivax* were hypnozoites inside infected-hepatocytes (Krotoski, 1985). As this liver stage cannot be detected using currently available diagnostic methods, it constitutes a silent reservoir of the disease and a major threat for malaria elimination. However, as recently reviewed (Fernandez-Becerra et al., 2022), new evidence indicates that cryptic niches outside the liver, in particular in the bone marrow (BM) and the spleen, represent a major source of hypnozoites-unrelated recrudescence. Thus, early studies on a clinical case of spontaneous spleen rupture revealed the presence of large numbers of intact parasites in the red pulp (Machado Siqueira et al., 2012). This observation raised evidence of a long standing hypothesis of spleen cytoadherence by reticulocyte-prone malaria parasites (del Portillo et al., 2004). Remarkably, recent studies of spleen rupture in Timika, Indonesia, have unequivocally demonstrated that the largest parasite biomass of *P. vivax* during chronic asymptomatic infections is observed in the reticulocyte-rich spleen (Kho et al., 2021a) (Kho et al., 2021b). Also, *P. vivax* parasites have been suspected to reside in the BM during chronic asymptomatic infections as originally observed in the late 19^th^ century (Bignami, 1894). Morphological and molecular evidence of the parasite in this hemopoietic tissues have been recently observed in patients (Baro et al., 2017; Brito et al., 2020). Noticeably, all patients presented defects in erythropoiesis and global transcriptional analysis corroborated these morphological observation (Baro et al., 2017; Brito et al., 2020). All together, these data call for a new paradigm in *P. vivax* research which should now incorporate these cryptic infections into its life cycle (**Figure 1**). This will clearly require a renewed emphasis on understanding the origin and significance of these cryptic erythrocytic niches, if elimination of malaria is to be achieved.

For ethical and technical reasons, working with the human spleen have been mostly limited to post-mortem analysis (Imbert et al., 2009) and studies on bone marrow aspirates are limited as it is considered a highly invasive clinical procedure. Here, we will review new enabling technologies on humanized mouse models and organs-on-a-chip, promising approaches to unravel the mechanistic particularities of these largely unexplored cryptic infections of *P. vivax*.

## Humanized Mouse Models

The interest in engrafting human immune systems to immunocompromised mice started with the discovery of the nude mouse in the laboratory of Dr. N.R. Grist (Flanagan, 1966). However, this early mouse strain was unable to sustain the engraftment of human bone marrow cells, thus failing to establish their growth (Ganick et al., 1980) (Macchiarini et al., 2005). A breakthrough came with the discovery of immunodeficient mice with the Prkdc-scid mutation, which in humans is called Severe Combined Immunodeficiency (SCID) resulting in the C.B-17-Prkdc^scid^ mouse (Pearson et al., 2008). The severe combined immunodeficiency (SCID) is a loss-of-function mutation, which affects the protein kinase, DNA-activated catalytic polypeptide (PRKDC), leading to an inefficient DNA non-homologous end joining repair that is required for T cell and B cell receptor rearrangement, resulting in a lack of mature T and B cells (Blunt et al., 1995) (Bosma et al., 1983) (Kirchgessner et al., 1995) (Mosier et al., 1988). Comparing SCID mice with nude mice there is a clear reduction of immune responses whereas the residual immune responses in nude mice are higher. Thus, SCID mice have been extensively used as recipients for human cell and tissue xenografts *in vivo*.

Another genetic deficiency that leads into a reduction of an adaptive immune response is the mutation in one of the recombination-activating genes Rag1 and Rag2, responsible for T cell and B cell receptor rearrangements (VDJ recombination), thus leading to lack of B and T cells (Shinkai et al., 1992). Rag-knockout (Rag KO) strains of mice are also used as recipients for human cell xenografts. These immunodeficient mouse strains are very useful as human cell transplant recipients, but they have several limitations, including a small production of T and B cells in older SCID mice and an increased susceptibility of SCID mice to preconditioning by radiation due to inefficient DNA repair. Moreover, there is still high levels of innate immune cells, which includes substantial NK cells that complicate their use in long-term studies and systemic reconstitution with human cells (Murphy et al., 1987).

An important advancement in the development of more immunodeficient mouse strains was the production of murine strains that have a mutation or deletion on the interleukin (IL)-2 receptor common γ chain (Il2rg or γc). The IL-2 receptor common gamma chain is an important cytokine receptor subunit for IL-2, IL-4, IL-7, IL-9, IL-15 and IL-21 and is indispensable for high-affinity binding and signaling of these cytokines (Cao et al., 1995) (DiSanto et al., 1995) (Ohbo et al., 1996). This gene disruption results in critical cytokine signaling networks that are required for both adaptive and innate immune responses leading to the lack of NK cells characterized by the absence of IL-15 signaling (Kennedy et al., 2000) (Kerre et al., 2002) (Ranson et al., 2003) (Traggiai et al., 2004) (Walsh et al., 2017). However, there is still some remaining phagocytic activity which triggers-on the development of graft-versus-host disease (GVHD) after the engraftment of human cells (Greenblatt et al., 2012).

The Cluster of Differentiation 47 (CD47) acts as a marker of self (Oldenborg et al., 2000), and its interaction with the inhibitory receptor signal regulatory protein α (SIRPα) on macrophages delivers a “do not eat-me” signal preventing phagocytosis of self-cells by macrophages. Therefore, if this interaction is not avoided on the murine environment, mouse *SIRPα* receptors will not recognize human CD47 and human engrafted cells will be phagocytized by murine macrophages. Fortunately, a polymorphism in the mouse *Sirpα* gene found in the non-obese diabetic genetic background (NOD) resembles to the human SIRPA gene, thus achieving phagocytic tolerance by the interaction *via* CD47- *SIRPA* (Yamauchi et al., 2013). Phagocytic tolerance can also be achieved by the transgenic expression of mouse CD47 on human hematopoietic cells (Takenaka et al., 2007; Legrand et al., 2011). On the other hand, phagocytic tolerance can be achieved temporarily by eliminFortunately, a polymorphism inating recipient phagocytic cells using clodronate-containing liposomes (Clo-lip), small hydrophilic molecule ingested by a macrophage in a liposome-encapsulated form. It will be accumulated within the cell as soon as the liposomes are digested with the help of its lysosomal phospholipases. At a certain intracellular clodronate concentration, the macrophage is eliminated by apoptosis. Alternatively, if phagocytic cells develop in an environment without CD47, then they become tolerized to cells that lack CD47 (Wang et al., 2007). Natural killer cells also have limited activity in NOD mice because of a defect in the NKG2D receptor (Ogasawara et al., 2003). All these properties have made the NOD background preferred for the development of models that receive xenotransplantation of cells and tissues from human origin.

The combination of IL2rg knockout with SCID or Rag KO mutations leads to highly immunodeficient strains that have neither mature T and B cells, nor NK cells, with severely debilitated monocyte/macrophage function. Reported strains that use IL2rg mutation are the NSG (Blunt et al., 1995; Kirchgessner et al., 1995), NOG (Blunt et al., 1995; Kirchgessner et al., 1995), NRG, and BRG (Shultz et al., 2007; Shultz et al., 2012; Akkina et al., 2016) models. Taking into account this mutation, these strains can support more efficient, long-term, stable, and systemic engraftment with human cells and tissues. Additional mutations in cytokine genes, the transgenic expression of human cytokines by exogenous plasmid injection, recombinant protein injection or the expression of additional transgene can help on the development of a specific cell type of interest. Yet, most strains support humanization of lymphoid and not myeloid erythropoietic lineages, thus limiting their use for studies on asexual blood stages in human malaria.

Noticeably, NSGW41 or NBSGW strains were created to overcome a lack of erythropoiesis and megakaryopoiesis in humanized mouse models. These strains support long-term engraftment of human hematopoietic stem cells (HSCs) without previous pre-conditioning therapy due to the loss of endogenous Kit function in Kit^W-41J^ allele (Yurino et al., 2016). This KIT-deficient mice demonstrated improved erythropoiesis formation into NOD/SCID/Il2rg^-/-^ (NSG) background (Cosgun et al., 2014; McIntosh et al., 2015; Rahmig et al., 2016). After reconstitution, significant numbers of mature thrombocytes were present in the peripheral blood while human erythroblasts were seen in the bone marrow (BM). In addition, the morphology, composition, and enucleation ability of *de novo* generated human erythroblasts were similar with those in human BM (Rahmig et al., 2016). After humanization of unconditioned NSGW41 or NBSGW mice, no human red blood cells (huRBCs) or lower numbers are detected in circulation, whereas the BM is highly repopulated with human erythroid progenitor cells, suggesting that human HSC engraftment supports an increased erythroid lineage production. All differentiation stages of human erythroid precursors have been detected and increased numbers of huRBC progenitors are present suggesting that human erythropoiesis and differentiation up to nucleated erythroid progenitor stages is supported by the murine microenvironment in these mouse strains (McIntosh et al., 2015; Rahmig et al., 2016). The enucleation frequency *in vivo* is totally different between humanized mice and human BM due to the formation of erythroblastic islands, which probably requires factors that are incompatible between both species (Dzierzak and Philipsen, 2013). Checking the expression of transcripts encoding for adult-type α-, β-, γ- and δ-globin in NSGW41 model showed that there is no block in human erythrocyte maturation and give paucity of huRBCs to either insufficient *in vivo* enucleation or SIRPα-independent phagocytosis (Rahmig et al., 2016). NSGW41 and NBSGW strains are thus an interesting model to study *P. vivax* blood stages in the bone marrow during infection since these are able to sustain human eythrocytic precursors by the engraftment with HSCs and can be maintained for longer periods of time if clodronate liposomes are administered depleting the remaining murine macrophages.

### Humanized Mouse Models in Malaria

#### Pre-Erythrocytic Infections

The pre-erythrocytic cycle of *Plasmodium spp.* infection begins when salivary gland sporozoites enter the human body through the bite of female infected *Anopheles spp.* mosquitoes during blood meals. Sporozoite then cross the skin barrier to enter the bloodstream before homing to the liver initiating asexual differentiation to form liver-stage schizonts. Upon completion of liver-stage development, thousands of merozoites are released into the blood circulation starting erythrocytic infections. Noticeably, in the case of *P. vivax* and *P. ovale*, a proportion of sporozoites enter a quiescent stage, the hypnozoite, after hepatocyte invasion. Without including the 8-aminoquinoline hypnozoitocidal drug in the standard anti-malarial treatment, the hypnozoite remains in the liver causing relapses of the disease. These relapses accounted for over 70% of clinical cases in endemic areas (Commons et al., 2020) causing significant morbidity and sustaining the transmission cycle. Primaquine and tafenoquine have been proven to effectively eradicate hypnozoite (John et al., 2012; Commons et al., 2018; Llanos-Cuentas et al., 2019) but the liabilities on the 14-days treatment regimen (primaquine) and the risk of hemolytic anemia in G6PD deficient patients have to be overcome. Therefore, there remains a major need of new drugs.

The lack of *in vivo* models to study liver-stage has been an obstacle for studying the biology of hypnozoite formation and drug development. Indeed, as detailed below, this was only possible through the recent development of humanized mouse models engrafted with human hepatocytes. Interestingly, this mouse liver repopulation with human hepatocytes required the establishment of two premises: (i) an immuno-compromised recipient mouse and (ii) the initiation of liver injury that depletes mouse hepatocytes, thus creating a niche that allows human hepatocytes to repopulate the mouse liver. Currently, there are three humanized mouse models that can be repopulated with human hepatocytes and have been mostly used to study *Plasmodium* infections:

The first liver-humanized model described was the albumin-urokinase-type plasminogen activator (Alb-uPA) transgenic SCID mouse in which the urokinase transgene is linked to an albumin promoter (Mercer et al., 2001). This resulting in the elevated level of plasma uPA, hypofibrinogenemia and accelerated hepatocyte cell death causing sub-acute liver failure. The transplantation of human hepatocyte into 7-12 days Alb-uPA mouse allow a high repopulation of human hepatocytes resulting in human/mouse chimeric liver with 60-70% observable human hepatocytes. The Alb-uPA human liver chimeric SCID mouse model (Alb-uPA huHep mouse) has been shown to support *P. falciparum* infection (Sacci et al., 2006). One disadvantage of this humanized mouse model is the continuous expression of uPA transgene that causes a progressive damage to the liver parenchyma probably *via* activation of plasminogen, which regulates the activity of matrix metalloproteinases that are critical for liver cell growth. Therefore, Alb-uPA model/uPA-dependent models have a limited utility for many applications due to this disadvantage, as well as a few others such as very poor breeding efficiency, renal disease, and a very narrow time window for transplantation before the mice submit to their bleeding (Heckel et al., 1990).

After Alb-uPA huHep mouse model, the FRG KO mouse model with triple knock out of tyrosine catabolic enzyme fumaryl-acetoacetate hydrolase FAH, Rag2^-/-^ and IL2rγ^null^ was developed. The fumaryl-acetoacetate hydrolase (FAH) KO leads to the toxic accumulation of fumaryl-acetoacetate, an intermediate of the tyrosine hormone metabolism (Azuma et al., 2007) causing liver injury. Like in Alb-uPA model, the FRG KO model can be transplanted with human hepatocytes (changing its name to FRG KO huHep) (Azuma et al., 2007). These FAH KO mice maintain their hepatocytes only in the presence of 2-(2-nitro-4-trifluoromethylbenzoyl)-1,3-cyclohexanedione (NTBC) and lose them when the drug is withdrawn (Grompe et al., 1995). This model is also able to support complete development of *P. falciparum* liver-stage (LS) infections (Vaughan et al., 2012). Vaughan and collaborators have observed that backcrossing the FRG KO huHep model on the NOD background (FRG NOD mouse) results in the transition of exo-erythrocytic merozoites stage to blood stage infection (Vaughan et al., 2012). This model also supports successful transition of recombinant *P. falciparum* parasites from various experimental genetic crosses (Vendrely et al., 2020). The FAH KO mice also show some disadvantages including the development of liver carcinomas because of their type I tyrosinemia and the continued or intermittent drug treatment after humanization to suppress the development of liver cancer (Bissig et al., 2010). FRG KO huHep mouse model can also support complete development of liver-stage of *P. vivax* an importantly hypnozoite formation. (Mikolajczak et al., 2015) (Schafer et al., 2020).

The third liver chimeric humanized mouse model expresses the herpes simplex virus thymidine kinase (HSVtk) transgene under the control of a mouse albumin enhancer/promoter in the liver of NOG mice (TK-NOG) (Hasegawa et al., 2011). In this model, the HSVtk mRNA is selectively expressed causing severe parenchymal liver damage after ganciclovir (GCV) treatment which allows repopulation of human hepatocytes (Hasegawa et al., 2011). This TK-NOG huHep mouse model has demonstrated a normal systemic and metabolic function and can be maintained without administration of exogenous drugs. Noticeably, the humanized TK-NOG huHep mouse model maintains their synthetic function for a prolonged period of time (over 8 months), as well as very high plasma human albumin levels. Moreover, additional administration of GCV doses after engrafted with human hepatocytes enables the depletion of residual mouse hepatocytes after human cell reconstitution (Hasegawa et al., 2011). This model enables the study of *P. falciparum* and *P. ovale* liver-stage. Therefore, TK-NOG huHep mouse model may also serve as a suitable model for *P. vivax* liver-stage infection.

#### Erythrocytic Infections

Once the sporozoites invade and establish themselves in the liver, parasites undergo asexual multiplication and develop into schizont stages that finally generates exo-erythrocytic merozoites. Upon released, thousands of merozoites enter the bloodstream and initiate erythrocytic cycle. In bloodstream, some parasites undergo gametocytogenesis therefore developing sexual stages which can be transmitted to mosquito vector during the blood meal. The *in vivo* study of *Plasmodium* infection is more advanced in the FRG huHep mouse model. Injection of human reticulocytes/erythrocytes into the FRG huHep mouse resulted in successful transplantation of human blood generating FRG huHep-blood mouse model. The FRG KO huHep-blood mouse model has been shown to support *P. vivax* liver-stage and transition to blood-stage enabling *in vivo* drug efficacy testing (Mikolajczak et al., 2015). By injecting human reticulocyte on day 9 and day 10 post sporozoite injection, blood-stages of *P. vivax* were observed as early as 4 hours after reticulocytes injection. Thus, the model can be used for studying the liver stage-to-blood stage transition of P. vivax (Mikolajczak et al., 2015). The model has also been used to test the efficacy of a pre-erythrocytic vaccine against *P. vivax* by passive immunization of anti-PvCSP antibodies prior to sporozoite injection, demonstrating that the vaccine could be of high benefit by reducing the hypnozoite reservoir and thereby reducing the number of relapses (Schafer et al., 2021).

**Figure 2:**
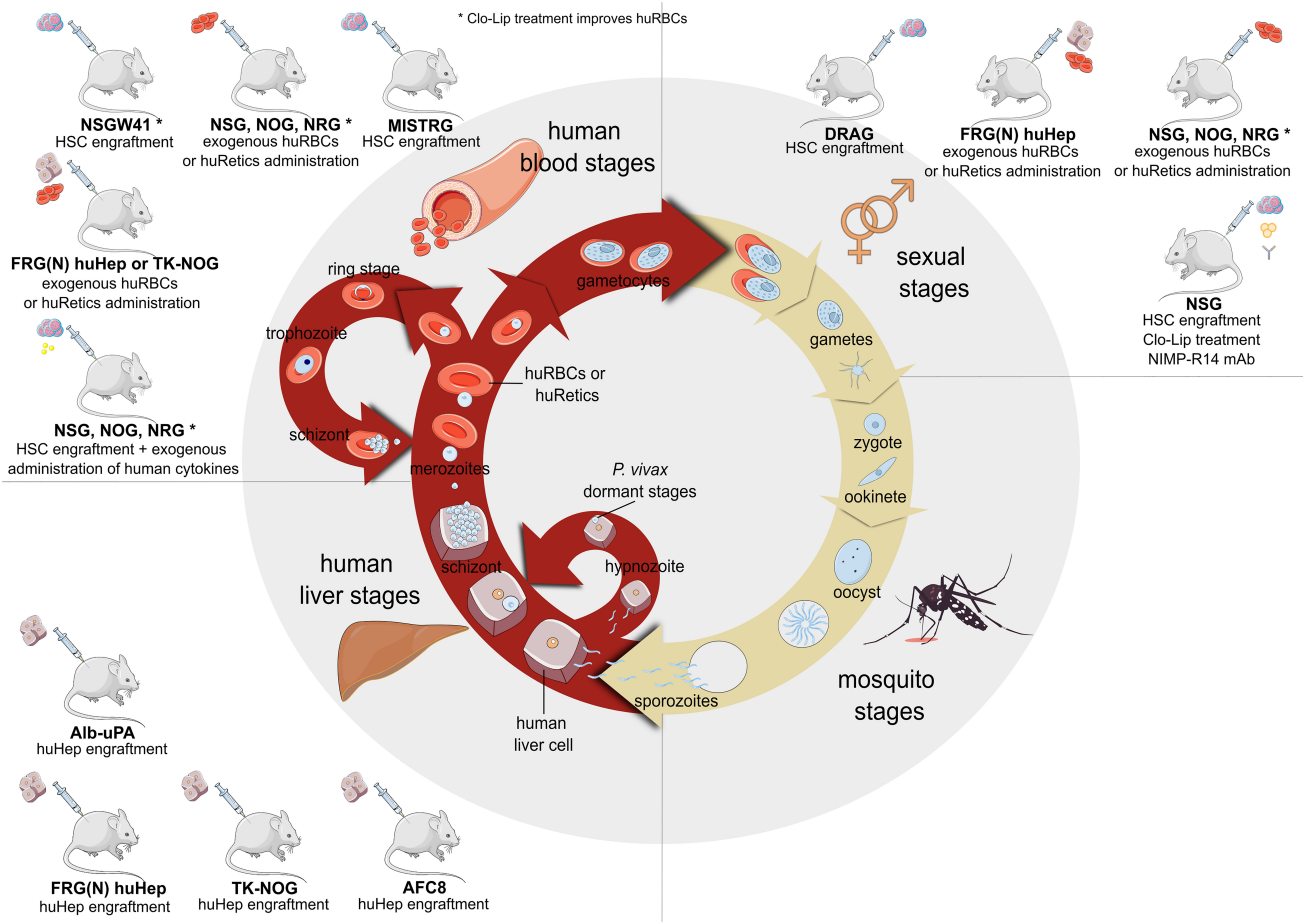
*Plasmodium falciparum* and *Plasmodium vivax* life cycle together with the currently available immunodeficient mouse models to study the different parasite stages. Models available to study liver stages are Alb-uPA, FRG (N) huHep, TK-NOG and AFC8 model (this last one has not already been tested for *Plasmodium* infections). Models to study blood stages are FRG (N) huHep model with exogenous huRBCs (for *Pf* infection) or huRetics (for *Pv* infection) administration; NSG, NOG and NRG models engrafted with human HSCs and treated with exogenous human cytokines or directly administered with exogenous huRBCs (for *Pf* infection) or huRetics (for *Pv* infection); NSGW41 (or NBSGW) and MISTRG models engrafted with human HSC (this last one has not already been tested for Plasmodium infections). Models to study sexual stages are DRAG model engrafted with human HSC, FRG (N) huHep model with exogenous huRBCs (for *Pf* infection) or huRetics (for *Pv* infection) administration, NSG, NOG and NRG models engrafted with exogenous huRBCs (for *Pf* infection) or huRetics (for *Pv* infection), NSG model engrafted with human HSC, treated with Clo-Lip and NIMP-R14 to deplete remaining murine neutrophils. *Clo-Lip treatment improves huRBCs engraftment/development. Human hepatocytes (huhep); human red blood cells (huRBC); human reticulocytes (huRetics); hematopoietic stem cells (HSC); Clodronate liposomes (Clo-Lip); *Plasmodium falciparum* (Pf); *Plasmodium vivax* (Pv); albumin-urokinase-type plasminogen activator (Alb-uPA); Knock-out (KO); FAH, Rag2^-/-^ and IL2rγ^null^ (FRG); thymidine kinase NOG (TK-NOG); NOD.Cg-Prkdc^scid^ Il2rg^tm1Wjl/^SzJ (NGS); NOD.Cg-Prkdc^scid^ Il2rg^tm1Sug/^JicTac (NOG); NOD.Cg-Rag1^tm1Mom^ Il2rg^tm1Wjl/^SzJ (NRG); C;129S4-Rag2^tm1.1Flv^ Csf ^tm1(CSF1)Flv^Csf2/Il3^tm1.1(CSF2,IL3)Flv^ Thpo^tm1.1(TPO)Flv^ Il2^gtm1.1Flv^ Tg(SIRPA)1Flv/J (MISTRG); NOD.Cg-Kit^W−41J^ Prkdcscid Il2rg^tm1Wjl/^ WaskJ (NSGW41); HLA-DR4 Rag^-/-^ IL2rg^-/-^ NOD (DRAG). (Created with Inkscape by Iris Aparici-Herraiz).

The FRG huHep mouse model has been improved recently (Schafer et al., 2020) by combining an immunomodulatory treatment in which Clodronate liposomes and Cyclophosphamide were co-administered to deplete murine macrophages and neutrophils, respectively (Foquet et al., 2018). This help to increase the lifespan of the infused human reticulocytes in the so called FRGNKOhuHep/huRetic mouse model. Blood chimerism reached 30% after the second infusion of reticulocytes. Liver stages development in this mouse model resulted in the release of merozoites which were able to invade the infused human reticulocytes starting on day 9 onwards with the highest blood parasitemia on day 10 post sporozoite injection. This procedure allows efficient and reproducible transition of *P. vivax* liver-stage to blood-stage and specially gametocyte. The FRGNKOhuHep/huRetic model allows the study of vaccine candidates for the blockage of blood stages (Schafer et al., 2020). Moreover, in FRGNKOhuHep/huRetic model was detected a subset of exo-erythrocytic schizonts expressing the sexual stage marker Pvs16 as early as 2 days after the beginning of blood stage infection indicating that exo-erythrocytic merozoites might be pre-programmed to become gametocytes in the first cycle of blood stage infection. However, the mature gametocyte marker Pvs230 was not detected. Therefore, FRGNKOhuHep/huRetic model allows the natural route of infection, liver-stage development and transition to blood-stage, providing a valuable system to test liver- and blood-stage vaccines and drug candidates (see **Figure 2**).

### Perspectives and Challenges of Humanized Mouse Models for Studying *P. vivax* Malaria

Advances on humanized mouse models are leading to a better understanding of malaria parasite biology, pathogenesis, and immunology as well as allowing testing, discovery and validation of new drugs and antigens for vaccination. Therefore, humanized mouse models can be seen as the link between rodent models and human infections to translate knowledge from both. In the case of *Plasmodium vivax*, this is of outmost importance as this species lacks a continuous *in vitro* culture system for blood stages (Noulin et al., 2013) and, in addition to the liver, the bone marrow and spleen have recently shown to represent a large biomass of hidden parasites in natural infections. In that sense, the generation of FRGNKOhuHep/huRetic model represented a major breakthrough for the study of *P. vivax* liver stages and its transition to blood stages since this model can sustain both stages, as well as, the possibility to study gametocytogenesis since sexual stage marker Pvs16 was early identified in the onset of the infection (Schafer et al., 2020). However, next-generation humanized mouse models for vivax malaria research will require humanization of the liver, bone marrow and spleen for studies on cryptic pre-erythrocytic and erythrocytic infections. Unfortunately, access to human fetal tissues and broad availability to the research community will limit their use. Therefore, novel enabling technologies reducing the use of animal experimentation and widely available, are also needed to advance research knowledge on cryptic pre-erythrocytic and erythrocytic infections.

## Organs-on-a-Chip

The development and implementation of two-dimensional (2D) cultures in cell biology has revolutionized our knowledge since the 19^th^ century description of frog embryos in hanging drops of coagulated frog lymph (Harrison et al., 1907) and the use of Petri dishes named after the inventor (Fischer, 1887). In 2D cultures, cells are grown as a monolayer under controlled physicochemical parameters such as oxygen, pH and temperature, as well as suitable growth medium, for trying to recapitulate as closely as possible the cellular microenvironment of the cells. 2D cell culture systems have advanced significantly our knowledge on cell biology and for many simple applications they will continue providing new insights into cell biology. However, as cells grow, their morphology changes by flattening and distorting as well as by forming a simple monolayer where they present forced, artificial polarization. Moreover, cells are subjected to excessive nutrition, molecular gradients cannot be reproduced and the characteristics of the extracellular matrix (ECM), including its architecture and stiffness, are also altered (Pampaloni et al., 2007). The simplicity of these traditional 2D culture systems, usually consisting of a single-cell type, makes them robust to perform high-throughput experiments; yet, they provide little information about complex systems such as tissues or organs, where cell-cell and cell-matrix communication is essential and where cellular *in vitro* mimicry of the micro-geometry of native *in vivo* environments is needed.

### Microfluidics Models

Back in the 70’s of last century, the revolution that supposed the use of the micro-fabrication methods from the industrial microelectronics applied to other materials, such as glass and polymers, lead to the development of miniaturized electro-mechanical components with sensors and acturators called Micro-ElectroMecanical Systems (MEMS). Noticeably, when MEMS were used to handle fluids, the term microfluidics was introduced (Whitesides, 2006). The main advantage of these devices relies on the capability to handle small fluid volumes, for instance a microchannel of hundreds of microns can hold fluid volumes at the nano liter range (100 x 100 x 100 μm = 1nL). This capability of generating controlled environments with low volume increased the processing velocity (Whitesides, 2006). Afterwards, microfluidics research centered on the development of analytical devices called Lab-on-a-Chip (LOC) and micro-Total analysis (µTAS) in order to develop point of care (POC) devices (Dittrich et al., 2006) (Yager et al., 2006). On these POC devices, researches were looking to develop fast, reliable and low cost diagnostics capable to process and analyze proteins, enzymes or cells (Yager et al., 2006).

Nowadays, it has been demonstrated that cell viability can be maintained in these micro systems with the appropriated ECM coatings, culture media and flow conditions. Then, as for the conventional *in vitro* models these cultured cells can be induced to express and maintain specific tissue functions in a controllable environment, with the capacity of replicating tissue/organ microstructures. These devices, now known as “organs-on-chips” (OOC) (Huh et al., 2011) (Hammel et al., 2021) (Moraes et al., 2012) (Bhatia and Ingber, 2014) are essentially, micro structured microreactors containing microchannels created on polymeric materials or glass that contain and compartmentalize cultured cells. The greater promise of these microsystems lies in the accuracy to recreate physical and biochemical microenvironments of specific and key compartments of living organs that are crucial for organ-level functions.

### Platform Fabrication

Soft lithography with PDMS is the most used technique for the Organ on a Chip platforms fabrication (Huh et al., 2011). This elastomeric material is cheap, transparent, gas permeable and biocompatible. However, PDMS absorbs hydrophobic molecules that could affect some analytical processes (Toepke and Beebe, 2006) that would be important for the final purpose of the OOC model. Therefore, this possibility should be taken in consideration, and, if needed be addressed by surface modification (Herland and Yoon, 2020) or by using alternative materials (Campbell et al., 2021).

### Adding Dimensionality

Over the last decades, the superiority of microfluidic-based cell culture platform has been demonstrated in comparison with static 2D culture in regard to the growth of cells and specific physiological functions (Jang et al., 2015), (Terrell et al., 2020) (Chou et al., 2020). Indeed, microfluidic-based culture platforms allows a continuous supply of nutrients and oxygen while removing cells’ metabolic waste - maintaining a stable microenvironment - for optimal cell growth and function. Moreover, microfluidics allows cells to experience stable molecule gradients and shear stress mimicking the *in vivo* blood physics in microcapillaries. Interestingly, this approach has the inherent flexibility of allowing multiple human cell types cultured in distinct compartments to be connected for inter-tissue modelling obviating the inter-species discrepancies of animal models (Maschmeyer et al., 2015) (Picollet-D'hahan et al., 2021). Nevertheless, the first-generation microfluidics approach frequently cultured cells as a 2D monolayer. In this sense, these cell-on-a-chip approaches failed to recapitulate the complex 3D cell–cell and cell-ECM interactions observed at the *in vivo* microenvironments.

Accumulating evidence clearly highlights the importance of tridimensionality for *in vitro* cell culture. Indeed, cells *in vivo* are often surrounded by ECM, which is pivotal to maintain the *in vivo*-like cell behavior including cell polarity, proliferation, migration and gene expression (Malinen et al., 2014) (Fontoura et al., 2020). Thus, the successful development of Organ-on-a-chip models requires the culture of multiple cell types in a highly controlled 3D microenvironment. This highlights the importance of developing fully customizable 3D matrices that resemble the native environment of each cell type. In this sense, hydrogels emerge as an important tool to take microfluidic models to the organ level.

### Hydrogels for Tailored Organs-on-a-Chip

Hydrogels are polymeric materials of cross-linked hydrophilic macromolecules with the ability to retain high amounts of water. Importantly, several types of natural and synthetic hydrogels are available possessing a wide range of relevant biochemical and biophysical features including cytocompatibility, biodegradability, and viscoelastic properties (Neves et al., 2020). Moreover, the easy visualization of cells embedded within the hydrogel, often transparent, is a plus for the real-time monitoring of cell behavior, essential for any organ-on-a-chip approach. Notably, hydrogels need to be carefully selected and their inherent properties fine-tuned depending on the intended use. Natural hydrogels including Matrigel, collagen and alginate typically display milder gelling conditions compared to synthetic ones as polyacrylamide or polyethylene glycol (Hu et al., 2022). Indeed, most of 3D cultures have relied on the use of natural hydrogels (Santos Rosalem et al., 2020). Basement membrane-derived matrigel is possibly the most widely used basement membrane hydrogel in 3D organoid culture experiments (Rossi et al., 2018). Matrigel is composed by a plethora of different rat basement membrane components including laminin-1, collagen IV, entactin, and heparin sulphate proteoglycans, as well as numerous growth factors (Blondel and Lutolf, 2019). Nevertheless, several drawbacks of this hydrogel include (i) unquantifiable lot-to-lot variability which undermines inter-laboratory reproducibility, (ii) presence of ill-defined amounts of growth factors making this approach unsuitable for understanding cell signaling and (iii) their high susceptibility to protease-mediated degradation (impacting the matrix stability) which may often preclude long-term cell culture (Blondel and Lutolf, 2019). On the other hand, alginate-based hydrogels are non-adhesive biomaterials, as cells cannot establish specific attachment points with the polymer itself (Bidarra and Barrias, 2019) (Neves et al., 2020). Interestingly, this feature is rather useful in artificial microenvironment design as these materials can act as blank-slates. Importantly, natural alginate can be chemically modified to promote specific biological responses in a highly tunable fashion through the incorporation of moieties that specifically modulate cell-material interactions (Bidarra et al., 2011) (Caires et al., 2015). Indeed, depending on the intended need alginate has been shown to acquire key biological features as cell adhesiveness, guided differentiation and matrix proteolytic degradation through chemical modification with instructive peptides. These designer hydrogels have been shown to offer unprecedent control over the ECM viscoelastic and biochemical properties and cell fate (Bidarra et al., 2010; Bidarra et al., 2011; Fonseca et al., 2011; Maia et al., 2014). Collectively, this next generation of bioengineered organ-on-a-chip hold the promise to bridge even further the gap between standard 2D cell culture and the more complex, expensive and ethically controversial animal experimentation in malaria research.

Since the study of *P. vivax* infection encompasses the interrogation of their distinct stages over multiple organs, in the next section we will explore the minimal functional units of liver, bone marrow and spleen. Most importantly, we will then review the available *in vitro* models that attempt to recapitulate *P. vivax* life cycle in each of these organs. Finally, we will address the outstanding questions and challenges for the next generation of bioengineered 3D OOC models in *P. vivax* research.

### The Liver Niche

The liver is a major organ that performs more than 500 physiological functions including compound detoxification, decomposition of red blood cells and production of hormones (Calitz et al., 2018). The portal hepatic lobules are the minimal functional units of liver, which includes three classical hexagonal hepatic lobules, and are considered the structural unit of the liver. These structures are composed mostly by parenchymal cells hepatocytes (up to 60%) while the remaining includes non-parenchymal cells as hepatic stem cells, connective tissue cells, hepatic stellate cells, monocytic Kupffer cells and endothelial cells (Miyajima et al., 2014). The lobular acinus can be further divided into three main zones of decreasing oxygen tension due to increasing distance towards the radial hepatic arterioles (Panday et al., 2022). Most notably the liver zonation seems to display distinct hepatocyte metabolic profiles and functions. Nevertheless, their role in host-pathogen interaction remains unknown.

#### Biophysical Features of Native ECM

The native ECM composes up to 10% of the liver volume, providing structural support, and has a Young’s Elastic Modulus that ranges between 300 and 600 Pa (Wu et al., 2018; Passi and Zahler, 2021) to provide structural support. Most notably, it has been shown that a healthy liver ECM is composed by more than 150 different proteins, including the most abundant, fibronectins, elastin, and fibrillar collagens (Naba et al., 2014; Arteel and Naba, 2020).

#### Organ Infection by *P. vivax*

During *Plasmodium vivax* infection the female *Anopheles* inoculated sporozoites, will reach the liver *via* blood circulation and invade the target hepatocytes either through Kupffer cells or *via* sinusoid endothelial cells (Pradel and Frevert, 2001; Tavares et al., 2013; Venugopal et al., 2020). However, others claim that a direct invasion of hepatocytes by sporozoites is also possible (Frevert et al., 1993; Ejigiri and Sinnis, 2009). Within invaded hepatocytes these sporozoites rapidly proliferate and generate thousands of merozoites giving rise to schizonts. This parasitic intrahepatic cycle develops over 7 days until the rupture the liver schizonts from infected/dying hepatocytes (Venugopal et al., 2020). Importantly, this rupture mediates the release of merozoites into blood circulation, where they preferentially invade young reticulocytes to start the intraerythrocytic life cycle. In contrast to this route, some *P. vivax* sporozoites can undergo a non-proliferative, metabolically quiescent stage known as hypnozoites (Gural et al., 2018b; Sylvester et al., 2021). Most importantly, these hypothetical liver-dwelling hypnozoites may be reactivated months or years and are a major cause of clinical relapse in *P. vivax* (Markus, 2018; Taylor et al., 2019). Unfortunately, the current inability to perform long-term *in vitro* culture of *P. vivax* severely hampers the controlled interrogation of this parasitic dormancy and re-activation processes.

#### 2D Attempts to Replicate the Liver Stage

So far, most of the key experimental insights on malaria research was provided either by *in vivo* rodent models or by the *in vitro* 2D monoculture (Voorberg-van der Wel et al., 2020a). Interestingly, most of these studies use hepatoma-derived cell line model systems such as HepG2, Huh7 and HC-04 (Hollingdale et al., 1985; Kaushansky et al., 2015; Ribeiro et al., 2016; Tweedell et al., 2019). While this provides a constant and reproducible source of host cells, these largely differ from primary hepatocytes in key biological features for *Plasmodium* infection (Manzoni et al., 2017), including the lack of important hepatic receptors and functions, lower metabolic activity and high dependence on glucose uptake (Castell et al., 2006; Meireles et al., 2017; Tripathi et al., 2020). Additionally, the high proliferative capacity of these cell lines precludes their application in the study of human *P. vivax* and hypnozoite formation, which present prolonged development times (more than 7 days). Interestingly, most of these bottleneck´s issues that hampered the *in vitro* study of *P. vivax* liver stage seem to be partially overcome by the use of primary human hepatocyte (PHH) cells. Nevertheless, upon 2D culture, the PHH progressively de-differentiate, losing their hepatic *in vivo* phenotype (Du et al., 2006). Indeed, several strategies have been explored to counter PHH hepatic dedifferentiation through limited biomimicry of hepatic microenvironment. Some of these attempts include (i) media supplementation with molecules that inhibit TGF-β, Wnt, Notch and BMP de-differentiation hepatocyte signaling pathways (Xiang et al., 2019; Lucifora et al., 2020), (ii) coating of the 2D substrate with hepatic-like ECM components as collagens (Itsara et al., 2018; Roth et al., 2018), (iii) co-culture with non-parenchymal cells such as fibroblasts (March et al., 2013; Gural et al., 2018b), or (iv) the use of multiple bioengineering approaches to implement the proper 3D tissue architecture (Chua et al., 2019; Mellin and Boddey, 2020).

#### Available 3D Models in Malaria (Focus on *P. vivax*)

Pioneering work by Dembele et al. has shown that it was possible to extend the lifespan/viability of sporozoite-infected simian hepatocytes in culture, *via* co-culture with human hepatoma cells HepaRG (to compensate infected hepatocyte cell loss) in a sandwich culture system (Dembele et al., 2014). Most notably, the use of collagen I coating allowed hepatocytes anchoring to the bottom of the culture dish while matrigel was placed on top of the cultured cells to impact ECM dimensionality and cell polarization. Interestingly, the authors have demonstrated that this approach led to a higher *in vitro* infectivity of *P. falciparum* (40-50 times) and that *P. cynomolgi* sporozoites infection (simian surrogate of *P. vivax*) of hepatocytes developed into both large dividing, but also, in small nondividing forms of the parasite during 40 days of culture (Dembele et al., 2014). Collectively, the authors reported the completion of the full liver cycle of the parasite with development of schizonts, hypnozoites formation up to 15 days with subsequent functional re-activation in culture. Most recently, Voorberg-van der Wel et al. also demonstrated that *P. cynomolgi-*infected primary rhesus hepatocyte cultured in collagen-coated substrates could be routinely maintained for 3 weeks (Voorberg-van der Wel et al., 2020b). This feature was also achieved by Roth et al. with PHH-infected with *P. vivax* using the same commercially available system (Roth et al., 2018). Interestingly, it was shown that, under such conditions, it was possible to explore malaria hypnozoite reactivation *in vitro* using a dual fluorescent *P. cynomolgi* reporter line (Voorberg-van der Wel et al., 2020b).

Tissue dimensionality is a key factor for imposing the right cell polarity and functionality. In this sense, Arez et al. have employed a stirred tank culture system to generate spheroids of human hepatic cell lines with a stable hepatic phenotype up to four weeks. Interestingly, *P. berghei* invasion and development were recapitulated in these hepatic spheroids, yielding functional blood-infective merozoites (Arez et al., 2019). Most importantly, Chua et al. have demonstrated that the formation of spheroids of simian and PHH in the soft macroporous 3D Cellusponge platform, which resemble the native liver ECM, supported the complete liver stage life cycle of both *P. cynomolgi* and *P. vivax* parasites *in vitro* for up to 30 days (Chua et al., 2019).

The importance of heterotypic cell-cell interaction was illustrated by showing that a combination of PHHs with supportive fibroblasts in a 2D multiwell micropatterned co-culture (MPCC) format was able to stabilize *in vivo*-like hepatocyte-specific functions and metabolism up to 4-6 weeks (Gural et al., 2018b). Most interestingly, it was demonstrated that this MPCC platform was also able to support the full liver cycle of *P. vivax* including the formation and reactivation of hypnozoites *in vitro* (March et al., 2015; Gural et al., 2018b). In a similar multiwell-based approach, it was also highlighted that substrate stiffness can also play a role in maintaining PHH phenotype (Maher et al., 2020). Indeed, these authors obtained similar results for *P. vivax* infection by simply exploring the use of soft PDMS molds to emboss hepatocyte-confining microfeatures into standard culture polystyrene microplates. Although further improvements in terms of tissue engineering complexity (i.e. with integrated dimensionality, heterotypic co-culture, improved infection rate, among others) are required to resemble the *in vivo* situation, this type of systems provided the first steps towards the future *in vitro* study of the liver hypnozoite dormancy/activation of the human *P. vivax*.

### The Bone Marrow Niche

The bone marrow is located in the trabecular cavities of the long bones, pelvis, sternum, etc. and is composed by multiple cell types surrounded by an heterogeneous ECM within an intricate microvascular network (Crane et al., 2017). Most importantly, the BM microenvironment is responsible for the *de novo* generation of 5 × 10^11^ blood components per day, including platelets, immune cells and red blood cells (Nombela-Arrieta and Manz, 2017). This is achieved throughout distinct BM-niches in a highly regulated process known as hematopoiesis (Nombela-Arrieta and Manz, 2017). For this process several cell types including mesenchymal stem/stromal cells (MSCs), osteoblasts, endothelial cells and others articulate in exquisite niches - local microenvironments that maintain and regulate stem cell fate - to simultaneously support hematopoietic stem cell asymmetric division, progenitor cell proliferation and lineage commitment into the required blood components (Méndez-Ferrer et al., 2020) (Crane et al., 2017).

#### Biophysical Features of Native ECM

The BM can be functionally divided into 3 distinct regions: endosteal, central and perivascular niches (Nombela-Arrieta and Manz, 2017). Structurally, the endosteal niche is located in the vicinity of BM cortical bone and is composed by osteoblasts and osteoclasts and MSCs surrounded by a 35 to 40 kPa stiff ECM of collagen type I and IV, osteopontin and fibronectin (Nilsson et al., 2005; Engler et al., 2006). The central niche is mostly inhabited by adipocytes, macrophages and fibroblasts embedded in a softer 0.3 kPa ECM containing laminin, fibronectin, heparin, hyaluronic acid (Nilsson et al., 1998; Omatsu et al., 2010; Shin et al., 2014). This is followed by the perivascular niche with a reported ECM stiffness of 2-10 kPa composed of collagen IV, fibronectin, and laminin, where endothelial, stromal cells and MSCs are in close contact with the arterial and sinusoidal blood vessels (Siler et al., 2000; Nelson and Roy, 2016). As expected, the relative proximity of the niches with the vascular network creates particular oxygen gradients, decreasing oxygen tension from the perivascular to central and endosteal niches. This feature combined with niche-specific cytokine gradients are instrumental for the regulation of HSC function and quiescence (Spencer et al., 2014).

#### Bioengineered Organ Models

Not surprisingly, even with the recent improvements of 3D culture, we still fail to provide an adequate *in vitro* biomimicry of the complex nature of the bone marrow microenvironment (Raic et al., 2019). Several studies have faced difficulties to integrate the tissue-tissue interactions, biochemical gradients and mechanical forces acting in the distinct BM-niches (Santos Rosalem et al., 2020). Even so, the existing attempts to closely mimic BM niches are best provided by Organ-on-a-chip approaches. These microfluidic-based approaches can be broadly divided by the cell type (single (Sung and Shuler, 2009; Sung et al., 2010; Torisawa et al., 2016) vs co-culture (Carrion et al., 2010; Khin et al., 2014; Zheng et al., 2016)) and type of tridimensional ECM (Hydrogels (Thon et al., 2014; Torisawa et al., 2014; Bruce et al., 2015; Aleman et al., 2019; McAleer et al., 2019; Chou et al., 2020; Herland et al., 2020; Glaser et al., 2022) vs Scaffolds (Houshmand et al., 2017; Marturano-Kruik et al., 2018; Sieber et al., 2018) vs Cell-generated ECM (Zhang et al., 2014; Zhang et al., 2015; Wuchter et al., 2016)) used for the biomimicry of BM compartments.

Representing some of these examples, Chou et al. have reported a vascularized human BM-on-a-chip (BM chip) that supports the differentiation and maturation of multiple blood cell lineages, including reticulocytes, over 4 weeks (Chou et al., 2020). Interestingly, the authors demonstrate that this 3D fibrin/collagen hydrogel-based microfluidic culture system had vastly improved CD34^+^ cell maintenance and function over the standard 2D and 3D bulk approaches, modulating more accurately distinct aspects of hematopoiesis and BM pathophysiology. More recently, Glaser et al. has also demonstrated that it is possible to study niche-specific functions on BM pathophysiology using a 3D fibrin-based microfluidic co-culture system. For this, the authors have simultaneously used distinct chambers in the same chip that recapitulated either the endosteal and perivascular niches (Glaser et al., 2022). Most interestingly, this system had fully perfusable vascular networks that allowed the maintenance of CD34^+^ HSCs but also their proliferation and differentiation along myeloid and erythroid lineages with the release and the egress of neutrophils (CD66b^+^) through the microvascular network.

The described BM-on- a-chip systems have a wide range of applicability. Yet, none of these systems has been applied to the malaria research so far. Instead, the few reported studies in *P. vivax* research remain limited to address fundamental BM-related questions in 2D culture systems (Panichakul et al., 2007; Fernandez-Becerra et al., 2013; Martin-Jaular et al., 2013; Noulin et al., 2014).

### The Spleen Niche

The spleen is the largest secondary lymphoid organ which is primarily responsible for blood immune-surveillance and erythrocyte recycling (Bowdler, 2002) (Mebius and Kraal, 2005). The spleen’s architecture is composed by two minimal functional units, the white pulp and red pulp regions, which are connected by a marginal zone. The white pulp encompasses nearly 25% of the splenic tissue and is composed by lymphoid tissue (Kashimura, 2020). Here, two regions can be distinguished: (i) the periarteriolar lymphoid sheaths which have mostly T cells lining the central arteriolar surrounded by (ii) the lymph follicles where B cells divide and maturate (Bowdler, 2002). These regions are followed by a marginal zone composed by antigen presenting cells (dendritic cells and macrophages) which are in close proximity to the red pulp. The red pulp represents up to 75% of the splenic tissue and is formed by reticular cells and immune cells (granulocytes and monocytes/macrophages) (Bowdler, 2002). Importantly, the circulatory network in this region contains open spaces with a complex reticular mesh known as splenic cords. Before exiting this compartment the cells and RBCs pass through 1–2 µm open interendothelial slits (IES), before re-entering into venous sinuses circulation (Bowdler, 2002). Importantly, during this stage the stiffer dysfunctional or infected RBCs are unable to squeeze through being cleared from circulation by resident macrophages (del Portillo et al., 2012).

#### Biophysical Features of Native ECM

Apart from identifying the splenic region by the residing immune populations in these niches, each of these compartments can also be distinguished by the type of reticular network organization and matrisome (Lokmic et al., 2008). Interestingly, despite several studies reported the macro-stiffness of a healthy spleen in the order of 15-20 KPa, the ECM stiffness attributed to each splenic niche remains largely unknown (Giuffrè et al., 2019) (Veiga et al., 2017).

#### Organ Function During *P. vivax* Infection

Not surprisingly, during the intra-erythrocytic life cycle of *P. vivax*, the spleen constitutes the main organ involved both in immune recognition and elimination of parasitized reticulocytes (del Portillo et al., 2012). Interestingly, recent data strongly suggests that besides facing destruction the *P. vivax* parasites can also be actively accumulating in this organ as part of their asexual blood stage lifecycle (Kho et al., 2021a). This would constitute an important cryptic niche/reservoir of the parasite. Indeed, several reports pinpoint the ability of *P. vivax* in modulating VIR proteins for enhanced parasitic cytoadhesion to human spleen fibroblasts (Fernandez-Becerra et al., 2020). Nevertheless, the questions remain on whether *P. vivax* cytoadhesive mechanisms can be used to home to these splenic cryptic niches, what is the nature of these niches and how do they enable parasite survival in such harsh lymphoid environment.

#### Available/Reported Models in Malaria

**Figure 3:**
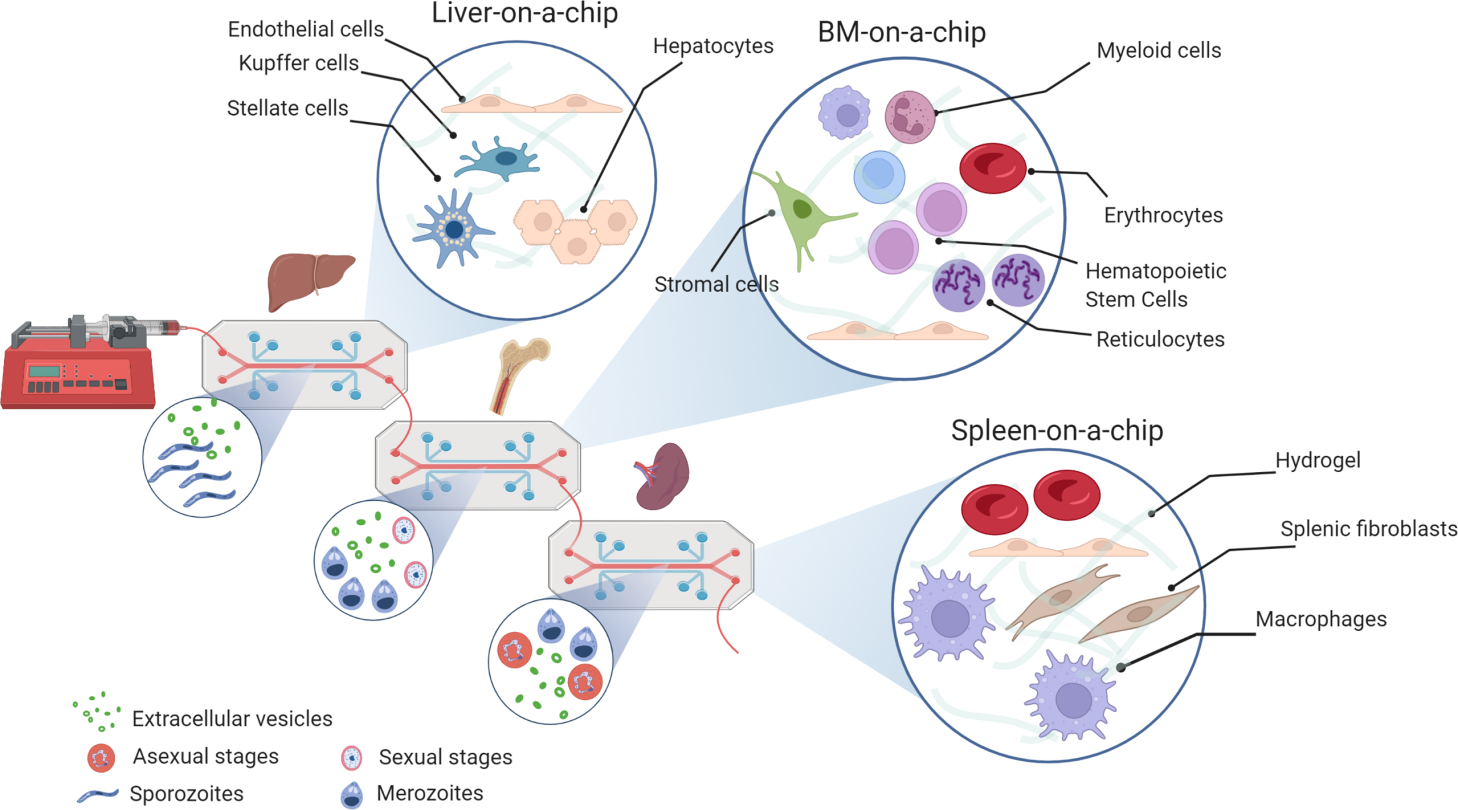
Organs-on-a-chip for cryptic infections in malaria research. Microfluidic system connecting the minimal functional units of a liver, bone marrow and spleen-on-a-chip on a PDMS support with controlled perfusion rate. Different molecular-design hydrogels can be used for tissue dimensionality, i.e., matrigel, alginate, fibrin/collagen. The small circles show the presence of malaria parasites and extracellular vesicles; the big circles show 3D cultures with illustrative examples of cell types present in each specific organ (Created with BioRender.com by Nuria Sima).

Unfortunately, our current understanding of *P. vivax*-infected RBCs interaction with the spleen derives mostly from post-mortem spleen sections (Machado Siqueira et al., 2012). (Kho et al., 2021a) (Kho et al., 2021b) (Imbert et al., 2009). One of the few examples that attempted to dissect the human parasitic infection in the human spleen was provided by Buffets’ group. Indeed, these authors have developed a technically challenging *ex vivo* perfused model of the human spleen that was able to maintain clearing functions of *P. falciparum*-infected RBCs for two hours experiments (Safeukui et al., 2008). Given the structural complexity of the splenic white pulp, no bioengineered *in vitro* 3D models were reported so far. Importantly, this precludes the comprehensive understanding of the human parasite-immune cell interactions occurring within this niche. Additionally, scarce literature exists regarding the *in vitro* biomimicry of the *Plasmodium*-infected erythrocyte clearance performed at the splenic red pulp interendothelial slits using a microfluidics-based approach (Rigat-Brugarolas et al., 2014) (Picot et al., 2015) (Guo et al., 2012) (Elizalde-Torrent et al., 2021) (see **Figure 3**).

### Key Challenges of OOC in Malaria Research

Understanding the interactions of malaria parasites during different life stages with the vascular endothelium under flow conditions is essential for unveiling pathophysiological mechanisms in malaria (Bernabeu et al., 2021). Initial examples of microfluidic devices containing endothelial cells cultured under flow conditions used commercial channel arrays. These approaches consisted on channel surface functionalization with collagen for allowing adherence and endothelization of these device structures with HUVEC cells (Introini et al., 2018). Recently, a vascular structure formed by endothelium pipes was also developed by culturing HUVEC cells on a microfluidic device that emulate arteriole, capillary and venule vessels geometry. Interestingly, the authors could monitor the spatial location and travel dynamics and the interaction of the infected red blood cells at different stages (Arakawa et al., 2020). These elegant studies demonstrated the proof-of-principle that OOC offer the potential of a transformative technology for studying malaria and other diseases. In fact, OOC are revolutionizing studies on physiopathology, drug development, and POC diagnostics in several different human diseases (Ingber, 2022).

In the case of *Plasmodium vivax*, OOC offers an unprecedented opportunity to study cryptic niches of infection, which perpetuate transmission and challenges malaria elimination. Noticeably, the liver, bone marrow and spleen are the organs where these hidden parasites reside making studies of these organs from natural infections, extremely challenging or not feasible. Previous development of a microscale human liver platform had demonstrated the feasibility of mimicking this organ in 2D to study human malaria liver infections (March et al., 2013). Later, the development of functional units of a liver-on-a-chip emulating the endothelial barrier of a liver sinusoid physically separated form primary human hepatocytes, showed the potential of using OOC of this organ in studies of physiology and drug-testing (Gural et al., 2018a).

In addition to latent liver infections, *P. vivax* evolved cryptic erythrocytic infections in the spleen where the largest parasite biomass is accumulated and in the bone marrow where sexual stages developed before reaching circulation (Fernandez-Becerra et al., 2022). The development of minimal functional units of these organs-on-a-chip thus offer another unprecedented opportunities to study these niches. Minimal functional units of the spleen-on-a-chip have been reported (Rigat-Brugarolas et al., 2014) (Elizalde-Torrent et al., 2021) (Picot et al., 2015); yet, in addition to rheological studies of infected blood they need now to incorporate ECM matrices and cells. In contrast, elegant OOC of the bone marrow showing sustain expansion of CD34^+^ cells, differentiation and egress of cells from the chip emulating vascular and bone marrow channels (Chou et al., 2020) (Glaser et al., 2022). It is therefore legitimate to speculate that human bone marrow and spleen OOC models will soon be applied to advance our knowledge of these cryptic erythrocytic infections and to screen for novel drugs as parasites in those niches seem to be shelter from antimalarial drugs (Lee et al., 2018).

As a *bona fide* aspect, OOC will also offer the opportunity to study the role of extracellular vesicles (EVs), nanovesicles of endocytic origin, in the formation/activation of such niches at a space and velocity mimicking functional units of these organs in 3D. Of note, *P. vivax* has a tropism for reticulocytes, young red cells residing in the bone marrow and the spleen, and which in their maturation to erythrocytes release EVs (Harding et al., 1983; Pan and Johnstone, 1983). Noticeably, circulating EVs from natural vivax infections have recently shown to contain parasite proteins and signal spleen fibroblast to increase surface expression of ICAM-1, thus facilitating cytoadherence of *P. vivax*-infected reticulocytes directly obtained from patients (Toda et al., 2020). EVs thus should contain precious insights into the formation of such niches.

## Concluding Remarks

Wrongly considered benign, vivax malaria has been a neglected disease. To further complicate matters, in spite of more than a century of research investigations, this species still lacks a continuous *in vitro* culture system for blood stages, which has severely hampered research of it. It is now clear that (i) *P. vivax* can cause severe disease (Baird, 2007), that (ii) chronic infections are associated with a higher risk of death than those caused by *P. falciparum* (Chen et al., 2016) and that (iii) *P. vivax* is a resilient species towards malaria elimination (Fernandez-Becerra et al., 2022). Most of this resilience relies on the fact that the largest parasite biomass resides in cryptic niches of the spleen, bone marrow and the liver. Many gaps in the knowledge of these niches as well as technical challenges to study them remain to be investigated and solved (**Box**). Yet, the use of humanized mouse models and organs-on-a-chip technologies reviewed here offer a technological breakthrough to study these niches, which ultimately might give new clues to develop the continuous *in vitro* culture system of *P. vivax*.

**Box:** Advances and challenges of humanized mouse models and organs-on-a-chip for malaria research.

Humanized Mice			Organs on a Chip	
Advances	Technical Limitations		Advances	Technical Limitations
Generation of immunocompromised mice to engraft human cells and tissues	– Incomplete mouse immunodeficiency hinders engraftment of human cells – Access to human fetal tissues – Minimal group size		Microfluidics	– Bubbles, fluidic connections, manipulation, tubbing and pumping systems must be robust and easy to use – Interconnection between different organ models to emulate tissue-tissue interaction.
Models for liver stages				
Alb-uPA	– Collateral effects due to the continuous expression of uPA transgene: Liver parenchyma damage, poor breeding efficiency, renal disease, narrow time window for transplantation		PDMS platform	– Absorption of hydrophobic molecules could be an issue for free vesicle circulation
FRG (N) huHep	– Liver carcinomas		Hydrogels Matrigel* *Alginate* *Fibrin/collagen*	– Ensure the homogeneous diffusion of oxygen, medium, blood, extracellular vesicles. – Emulate the mechanical properties of ECM of organs. – Allow internal vascularization and biochemical gradient formation. – Lot-to-lot variability – *Unsuitable for understanding cell signaling
TK-NOG				
Models for blood stages	– Incompatibility between human and mice factors leads to inefficient differentiation ofthe HSC			
NSG, NOG, NRG	– Dependence on human cytokines administration – Low capacity for human erythroid cells development			
NSGW41	– Human erythroid lineage engraftment in the bone marrow; yet, few or no red blood cells are observed in circulation		Organs-on-a-chip *BM-* *BM-on-a-chip* *Splenon-on-a-chip*	– Dependence on primary cells from human donors. – Donor-to-d onor variability – Emulate the mechanical properties of circulating blood. – Tissue complexity – Low *P. vivax* infection rate
FRG(N) huHep	– Periodical infusion of huRBCs or huRetics required			
TK-NOG				

Alb-uPA, albumin-urokinase-type plasminogen activator; KO, Knock-out; FRG, FAH, Rag2^-/-^ IL2rγ^null^; TK-NOG, thymidine kinase NOG; NGS, NOD.Cg-Prkdcscid Il2rgtm1Wjl/SzJ; NOG, NOD.Cg-Prkdcscid Il2rgtm1Sug/JicTac; NRG, NOD.Cg-Rag1tm1Mom Il2rgtm1Wjl/SzJ; NSGW41, NOD.Cg-KitW−41J Prkdcscid Il2rgtm1Wjl/WaskJ; hHSCs, human Hematopoietic Stem Cells; huRBCs, human Red Blood Cells; huRetics, human reticulocytes; PDMS, Polydimethylsiloxane; MPCC, come micropatterned cocultures; BM, bone marrow.

## Author Contributions

Wrote the manuscript: IAH, HC, HP. Contributed to the final manuscript editing: OC-F, NS, LM-M, RR, JS, WR, AH-M, CF-B, CB. Figures have been idealised and made by CF-B (**Figure 1**), IA-H (**Figure 2**), and NS (**Figure 3**). All authors reviewed the manuscript and approved the submitted version.

## Funding

IA-H is a predoctoral fellow supported by the Ministerio de Economia y Competitividad (FPI BES-2017081657). Work on humanized mouse models is funded by the Ministerio de Ciencia e Innovación (PID2019-111795RB-I00) and on Organs-on-a-Chip by “la Caixa” Foundation (LCF/PR/HR21/52410021). “This research is part of the ISGlobal “Centro de Excelencia Severo Ochoa 2019-2023” Program (CEX2018-000806-S) and of the “Molecular Mechanisms of Malaria Program” partially funded by Fundación Ramón Areces. ISGlobal and IGTP are members of the CERCA programme, Generalitat de Catalunya.

## Conflict of Interest

The authors declare that the research was conducted in the absence of any commercial or financial relationships that could be construed as a potential conflict of interest.

## Publisher’s Note

All claims expressed in this article are solely those of the authors and do not necessarily represent those of their affiliated organizations, or those of the publisher, the editors and the reviewers. Any product that may be evaluated in this article, or claim that may be made by its manufacturer, is not guaranteed or endorsed by the publisher.
